# Production of Volatile Moth Sex Pheromones in Transgenic *Nicotiana benthamiana* Plants

**DOI:** 10.34133/2021/9891082

**Published:** 2021-10-12

**Authors:** Rubén Mateos-Fernández, Elena Moreno-Giménez, Silvia Gianoglio, Alfredo Quijano-Rubio, Jose Gavaldá-García, Lucía Estellés, Alba Rubert, José Luis Rambla, Marta Vazquez-Vilar, Estefanía Huet, Asunción Fernández-del-Carmen, Ana Espinosa-Ruiz, Mojca Juteršek, Sandra Vacas, Ismael Navarro, Vicente Navarro-Llopis, Jaime Primo, Diego Orzáez

**Affiliations:** ^1^Institute for Plant Molecular and Cell Biology (IBMCP), Consejo Superior de Investigaciones Científicas (CSIC) - Universidad Politécnica de Valencia (UPV), Valencia, Spain; ^2^Jaume I University, Castellon de la Plana, Spain; ^3^Department of Biotechnology and Systems Biology, National Institute of Biology, Ljubljana, Slovenia; ^4^Jožef Stefan International Postgraduate School, Ljubljana, Slovenia; ^5^Centro de Ecología Química Agrícola, Instituto Agroforestal del Mediterráneo, Universitat Politècnica de València, Valencia, Spain; ^6^Ecología Y Protección Agrícola SL, Valencia, Spain

## Abstract

Plant-based bioproduction of insect sex pheromones has been proposed as an innovative strategy to increase the sustainability of pest control in agriculture. Here, we describe the engineering of transgenic plants producing *(Z)*-11-hexadecenol (Z11-16OH) and *(Z)*-11-hexadecenyl acetate (Z11-16OAc), two main volatile components in many Lepidoptera sex pheromone blends. We assembled multigene DNA constructs encoding the pheromone biosynthetic pathway and stably transformed them into *Nicotiana benthamiana* plants. The constructs contained the *Amyelois transitella AtrΔ11* desaturase gene, the *Helicoverpa armigera* fatty acyl reductase *HarFAR* gene, and the *Euonymus alatus* diacylglycerol acetyltransferase *EaDAct* gene in different configurations. All the pheromone-producing plants showed dwarf phenotypes, the severity of which correlated with pheromone levels. All but one of the recovered lines produced high levels of Z11-16OH, but very low levels of Z11-16OAc, probably as a result of recurrent truncations at the level of the *EaDAct* gene. Only one plant line (SxPv1.2) was recovered that harboured an intact pheromone pathway and which produced moderate levels of Z11-16OAc (11.8 *μ*g g^-1^ FW) and high levels of Z11-16OH (111.4 *μ*g g^-1^). Z11-16OAc production was accompanied in SxPv1.2 by a partial recovery of the dwarf phenotype. SxPv1.2 was used to estimate the rates of volatile pheromone release, which resulted in 8.48 ng g^-1^ FW per day for Z11-16OH and 9.44 ng g^-1^ FW per day for Z11-16OAc. Our results suggest that pheromone release acts as a limiting factor in pheromone biodispenser strategies and establish a roadmap for biotechnological improvements.

## 1. Introduction

Insect pheromones are a sustainable alternative to broad-spectrum pesticides in pest control. Different pheromone-based pest management approaches can be employed to contain herbivore populations, thus limiting damage to food, feed, industrial crops, and stored goods. These approaches include multiple strategies, such as (i) attract-and-kill strategies, in which pheromones are used to lure insects into mass traps; (ii) push-pull strategies, in which different stimuli are used to divert herbivores from crops to alternative hosts; and (iii) mating disruption techniques in which mating is prevented or delayed by providing males with misleading pheromone cues [1–4]. Broad-spectrum pesticides cause severe toxicity not only towards the targeted insect population but also towards predatory insects resulting in substantial ecological imbalances [5]. On the contrary, insect sex pheromones usually produced by females to attract males over long distances are highly species-specific and minimize environmental toxicity. Furthermore, pheromone-based pest control approaches are effective against pesticide-resistant insect populations and prevent the emergence of genetic pesticide resistance.

The global insect pheromone market was worth 1.9 billion USD in 2017, with projections reaching over 6 billion USD by 2025 [6]. Despite their biological potential and their value to farmers and the environment, their use suffers from some limitations: the chemical synthesis of insect sex pheromones can often be costly and complex and generate polluting by-products, which hamper their sustainability [7, 8]. The cost of chemically synthesized pheromones ranges from 500 to thousands USD kg^-1^, making this solution profitable only for very high-value end products [9]. To make pheromone production more sustainable, engineered biological systems can be designed to function as pheromone biofactories from which the molecule(s) of interest can be purified to formulate conventional traps [10]. Ideally, live biodispensers can be envisioned, which directly release pheromones into the environment in an autonomous, self-sustained manner [11].

Around 160,000 lepidopteran species and 700 lepidopteran pheromones are known [12–14]. Many of these moths are relevant for agriculture and rely heavily on pheromones for mating. Lepidopteran sex pheromones have been the focus of many attempts at biotechnological production, because of their relatively simple chemical composition and their economic relevance. Sex pheromones emitted by female moths are composed of a discrete blend of volatile compounds, mostly C10-C18 straight chain primary alcohols, aldehydes, or acetates derived from palmitic and stearic acids [15]. Although hundreds of species share the same pheromone compounds, the components of the pheromone blend and their relative abundance constitute highly precise, species-specific cues for mating. The biosynthesis of many moth pheromones shares three fundamental steps, which follow fatty acid biosynthesis. Fatty acid desaturases (FADs) introduce double bonds at specific positions in the carbon chain (the most common in Lepidoptera are *Δ*9 and *Δ*11). Fatty acyl reductases (FARs) produce fatty alcohols, with different substrate specificities (some accept only a limited range of substrates, while others are more promiscuous). Finally, aldehydes and acetates can be obtained, respectively, by oxidation and esterification of these fatty alcohols. In addition, other important modifications can occur before specification of terminal functional groups, especially chain elongation or shortening which, coupled with the desaturation steps, determine the structure of the carbon backbone [15]. The biosynthesis of the acetate esters is thought to be performed by acetyltransferases, although no insect acetyltransferases have been identified which work on fatty alcohols [9]. Acetyltransferases from other sources, like plants and yeasts, have nonetheless been discovered, which work efficiently on insect pheromone alcohols [16].

Plants represent an alluring platform to produce moth sex pheromones: the scalability and relatively low costs and infrastructure requirements of plant biofactories make this system versatile and sustainable. In plants, photosynthesis provides the precursors to start fatty acid biosynthesis in the chloroplast. In a pioneering study, Nešněrová et al. [17], took advantage for the first time of the plant fatty acid pool to produce lepidopteran pheromone precursors in plants. Later, in the most extensive screening of candidate genes so far, Ding et al. [18] identified the most effective among 50 different gene combinations to produce moth pheromones by transient expression in *N. benthamiana*. Subsequently, Xia et al. [19] established stably transformed *N. benthamiana* and *N. tabacum* lines expressing precursors for the synthesis of a wide range of moth pheromones. However, to date, no stable transgenic plants have been reported producing the actual volatile pheromone components.

In this work, we aimed to test the ability of *N. benthamiana* plants to act as constitutive moth pheromone biofactories. *Nicotiana* species (*N. tabacum* and *N. benthamiana*) are ideal chassis for metabolic engineering, due to their large leaf biomass (especially for plastid-derived products) and amenability to genetic manipulation, both through stable transformation and agroinfiltration. For stable pheromone production, we selected three of the genes identified by Ding et al. [18], namely, the *Amyelois transitella AtrΔ11* desaturase, the *Helicoverpa armigera* reductase *HarFAR*, and the plant diacylglycerol acetyltransferase *EaDAct* from the bush *Euonymus alatus*. The products of this pathway, *(Z)*-11-hexadecenol (Z11-16OH) and its ester *(Z)*-11-hexadecenyl acetate (Z11-16OAc), are components of the specific pheromone blends of almost 300 lepidopteran species [13]. The generation of transgenic pheromone-producing plants (originally named as “Sexy Plants”, SxP) turned out to be severely hampered by a strong growth penalty putatively imposed by the pheromone biosynthetic pathway. In the first round of attempts, only *N. benthamiana* plants accumulating the fatty alcohol were recovered, with all primary transformants showing dwarf phenotypes to different degrees. These transgenic lines were later shown to carry a truncated version of the *EaDAct* gene. This discovery led to the generation of new transformants, with new strategies aimed at ensuring the integrity of the construct that finally yielded a single transgenic line accumulating both the alcohol and the acetate components at relatively high levels, while maintaining acceptable levels of fertility and biomass production. This single line allowed us to gain insights into the challenges associated with fatty-acid derived pheromone production in plants, such as yield-associated growth penalties, changes in volatile profile, and compound volatility.

## 2. Results

### 2.1. Assembly of the Metabolic Pathway

**Figure 1:**
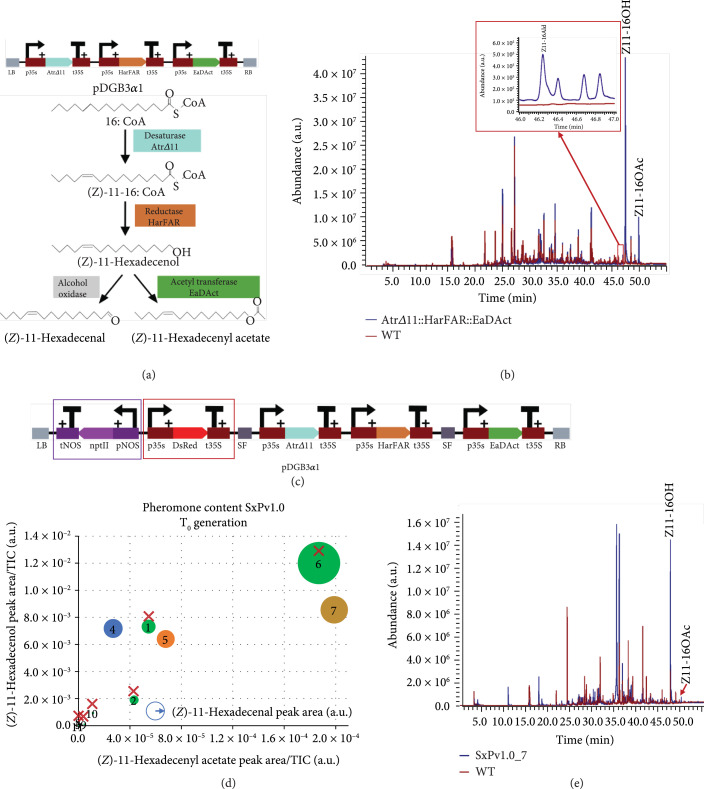
Stable and transient expression in *Nicotiana benthamiana* of the synthetic moth pheromone pathway. (a) Schematic view of the T-DNA construct used for transient expression, carrying the three transgenes *AtrΔ11*, *HarFAR*, and *EaDAct*, each under the control of the constitutive CaMV35s promoter and terminator, and the biosynthetic route of the moth pheromones. (b) GC/MS analysis of the volatile profile of *N. benthamiana* transiently expressing the transgenes (blue line) and a mock infiltrated plant with only P19 (red line). Peaks corresponding to the target insect pheromones are indicated with a label. Highlighted in red is the region of the (*Z*)-11-hexadecenal peak. (c) Schematic view of the T-DNA construct for the SxPv1.0 encoding the three transgenes and two selection markers. The two selection markers DsRed and NptII are highlighted in red and purple, respectively. (d) Pheromone content in SxPv1.0 T_0_ plants (numbered from 1 to 11). The diameter of each dot corresponds to the (*Z*)-11-hexadecenal level of each sample. Plants marked with a red cross died before seeds could be collected. (e) Overlapped chromatograms showing the volatile profile of a representative SxPv1.0 T_0_ plant (blue line) and a WT plant (red line).

To assess plant-based production of the target moth sex pheromone compounds (Z11-16OH and Z11-16OAc), a T-DNA construct encoding the three biosynthetic genes, each under the control of the constitutive CaMV35S promoter, was agroinfiltrated in plant leaves after being mixed in a 1 : 1 ratio with an *Agrobacterium* culture carrying the P19 silencing suppressor [20] (Figure 1(a)). The total volatile organic compound (VOC) composition was analyzed at 5 days postinfiltration by gas chromatography/mass spectrometry (GC/MS). GC peaks corresponding to the pheromone compounds Z11-16OH and Z11-16OAc were detected in samples transformed with all three enzymes, but not with P19 alone (Figure 1(b)). Moreover, both substances were among the most predominant compounds in the leaf volatile profile, indicating that the transgenes were expressed at high levels. Interestingly, a small peak identified as *(Z)*-11-hexadecenal (Z11-16Ald) was also detected in the agroinfiltrated samples, likely due to the endogenous activity of alcohol oxidases, as previously suggested by Hagström et al. [21]. This aldehyde is itself a component of the pheromone blends of around 200 lepidopteran species [13]. Based on these results, a multigene construct (GB1491) for stable transformation of *N. benthamiana* plants was assembled. This construct comprised the three constitutively expressed enzymes, the kanamycin resistance gene *NptII*, and the visual selection marker *DsRed* (Figure 1(c)). Plants resulting from this transformation were denoted as the first version of the pheromone-producing plant (SxPv1.0).

### 2.2. SxPv1.0 Stable Transformants

The transformation of *N. benthamiana* with the GB1491 construct resulted in the selection of 11 kanamycin-resistant shoots, which also showed red fluorescence resulting from the expression of *DsRed* (T_0_ generation SxPv1.0 plants). These shoots were further grown and rooted, and leaf samples were collected at the early flowering stage to assess pheromone production. As observed in Figure 1(d), several T_0_ plants presented detectable levels of all three pheromone compounds in variable amounts. The relative abundance of all three pheromone compounds was consistent in each plant, despite Z11-16OAc levels being much lower than expected in all cases compared to transient pheromone expression. Furthermore, although phenotypic evaluation of *N. benthamiana* T_0_ lines is generally cumbersome due to the influence of *in vitro* culture, severe growth penalties were observed in these plants, and only 5 out of 11 plants (SxPv1.0_4, 5, 7, 8, and 9) survived long enough to produce seeds.

**Figure 2:**
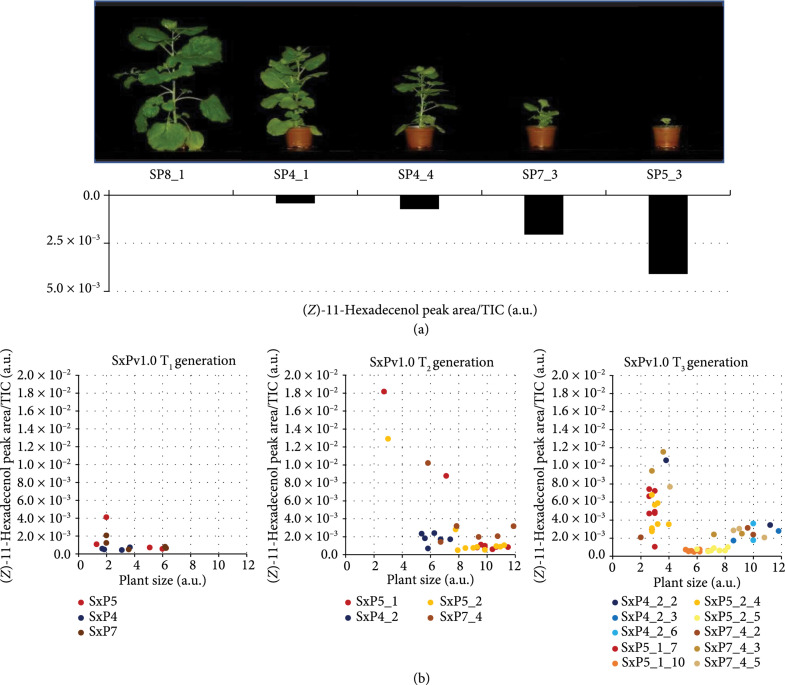
Growth penalty linked to pheromone production in SxPv1.0 plants. (a) Representative SxPv1.0 T_1_ plants with their corresponding (*Z*)-11-hexadecenol levels. (b) Correlation between (*Z*)-11-hexadecenol content (arbitrary units, a.u.) and plant size (a.u.) in all three generations of SxPv1.0.

To further understand the phenotypic effects of pheromone production, the progeny of plants SxPv1.0_4, SxPv1.0_5, and SxPv1.0_7 was analyzed up to the T_3_ generation and the plant size and pheromone production levels were recorded for each individual. In the T_1_ generation, growth penalties were also observed in most descendants for all three lines, generally associated with high pheromone production (Figures 2(a) and 2(b), A). Several plants could not be phenotyped, as they died soon after germination. Those producing enough seeds were brought to T_2_, where a similar trend was also observed (Figure 2(b), B). A few T_2_ plants clearly separated from the rest in terms of high Z11-16OH production, which was again associated with small size and reduced fertility. Neither T_1_ nor T_2_ plants showed signs of recovery in Z11-16OAc levels, although the corresponding GC/MS peak remained detectable and above the wild type (WT) baseline (not shown). At this stage, we decided to reevaluate the integrity of the T-DNA in T_2_ plants, finding that DNA rearrangements had occurred in all three lines in the *EaDAct* coding sequence, resulting in a truncated gene. Rearrangements and truncations of the T-DNA are not uncommon events in stable plant transformation [22, 23]. Interestingly, at least two independent truncation events could be inferred from PCR analysis of gDNA and cDNA samples. In SxPv1.0_7_4 plants, the presence of a ~700 bp insertion of a DNA fragment of plasmid origin could be identified at the 3${}^{\prime }$ end of the *EaDAct* coding sequence. In contrast, the same 700 bp genomic PCR fragment could not be recovered from the offspring of SxPv1.0_4_2, SxPv1.0_5_1, and SxPv1.0_5_2 plants, which nevertheless also had a truncated ORF, as evidenced by PCR analysis of cDNA samples (Figure S2). Despite the detection of a T-DNA truncation, the analysis of the SxPv1.0 offspring was continued up to T_3_ (Figure 2(b), C), where a sharp separation between low and high producers was consolidated. Interestingly, the offspring from the SxPv1.0_5_1_7 homozygous line (100% kanamycin resistant) comprised only high producer plants, whereas heterozygous lines as SxPv1.0_4_2_2 or SxPv1.0_7_4_3 segregated in high and low producers, these correlating with small and large sized individuals, respectively. This observation strongly indicates a drastic effect of transgene copy number on both growth and pheromone production.

### 2.3. New Stable Transgenic Versions SxPv1.1 and SxPv1.2

**Figure 3:**
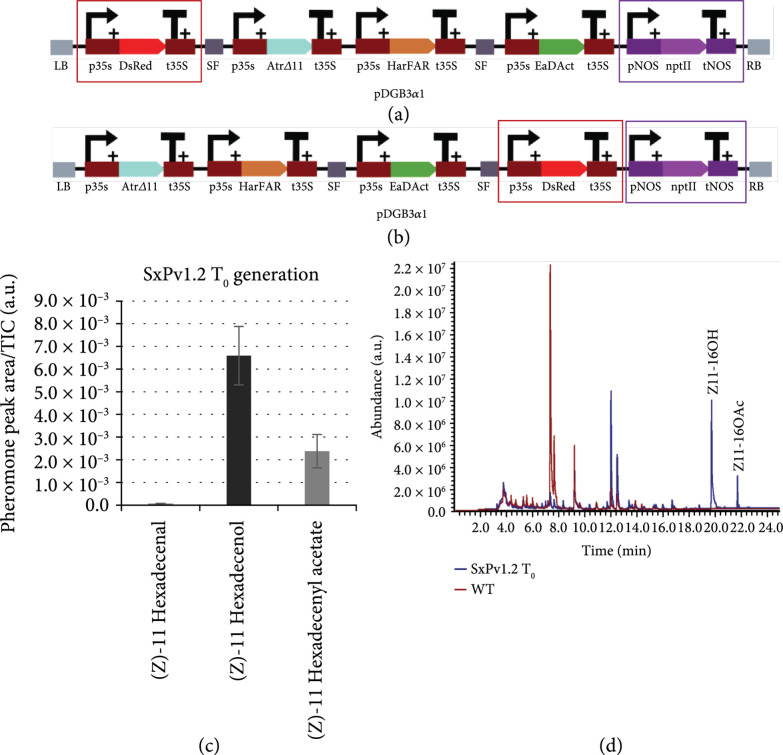
SxP version 1.1 and 1.2 stable plants. (a) Schematic representation of the T-DNA construct employed for stable transgenic SxPv1.1. The two selection markers *DsRed* and *NptII* are highlighted in red and purple, respectively. (b) Schematic representation of the T-DNA construct employed for stable transgenic v1.2. The two selection markers *DsRed* and *NptII* are highlighted in red and purple, respectively. (c) Pheromone content in the surviving SxPv1.2 T_0_ plant. Error bars represent the average±SE of 3 independent replicates. (d) Overlapped chromatograms showing the volatile profile of a SxPv1.2 T_0_ plant (blue line) and a WT *Nicotiana benthamiana* (red line).

The presence of at least two independent truncation events affecting *EaDAct* prompted us to design new transformation strategies by placing a selection marker adjacent to the *EaDAct* gene, ensuring its integrity. Two new DNA constructs were assembled (SxPv1.1 and SxPv1.2) carrying *DsRed* and *NptII* at different relative positions of the T-DNA, as depicted in Figures 3(a) and 3(b). Five SxPv1.1 and eight SxPv1.2 kanamycin-resistant T_0_ plants were recovered from each transformation, many of them showing detectable red fluorescence, but unfortunately all but one failed to produce detectable levels of pheromones. The only exception corresponded to plant SxPv1.2_4, which not only showed Z11-16OH and Z11-16Ald amounts comparable to the SxPv1.0 plants but also Z11-16OAc levels close to those measured in transient experiments (Figures 3(c) and 3(d)). SxPv1.2_4 presented premature flowering, a feature that is not unusual in T_0_*N. benthamiana* plants and produced viable seeds, giving us the opportunity to further investigate the phenotype of stable Z11-16OAc producers.

**Figure 4:**
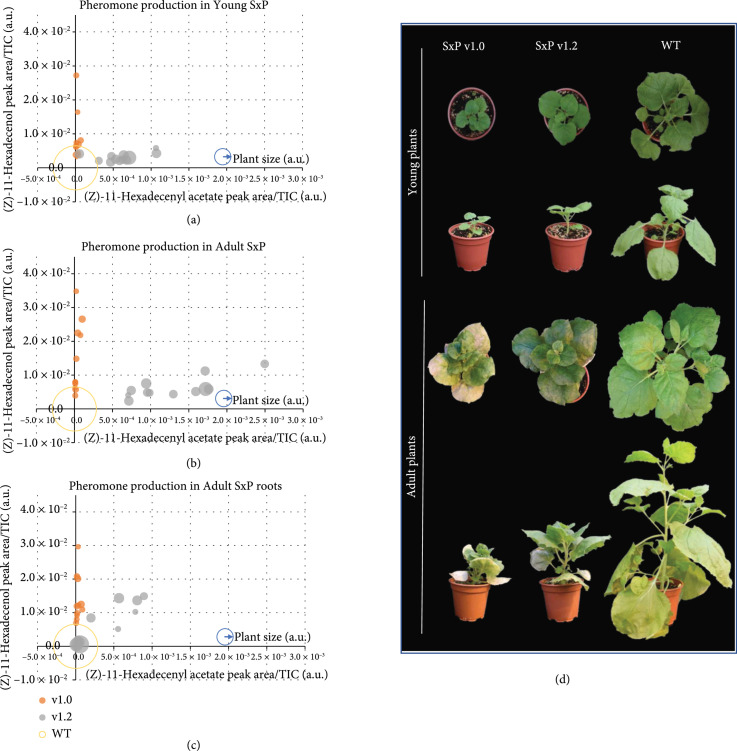
Comparative study between SxPv1.0 T_3_ and SxPv1.2 T_1_ plants. (a) Pheromone content in leaf samples from young plants of WT, SxPv1.0 and SxPv1.2 lines. (b) Pheromone content in leaf samples from adult plants of WT, SxPv1.0 and SxPv1.2 lines. (c) Pheromone content in root samples from adult plants of WT, SxPv1.0 and SxPv1.2 lines. The diameter of each dot corresponds to the plant size of each sample. Empty circles correspond to WT plants. (d) Comparative physiological development in SxPv1.0 5_1_7_X (T_3_), SxPv1.2 4_X (T_1_), and WT *Nicotiana benthamiana* plants at the young and adult stage. Pictures were taken from representative individuals at young (4 weeks after transplant) and adult (7 weeks after transplant) stages.

For a deeper understanding of the effect of fatty-acid-derived pheromone production in plant homeostasis, a comparative study between the progeny of the T_2_ SxPv1.0_5_1_7 homozygous line and the T_0_ SxPv1.2_4 line was performed. All analyzed SxPv1.2 T_1_ plants (>50) were kanamycin resistant, indicating multiple copy insertions. The relative levels of all three pheromone compounds in leaves at two different developmental stages (young and adult) were recorded for twelve T_1_ plants per genotype. Similarly, pheromone content in roots was also measured at the adult stage. Plant size was recorded for all analyzed individuals. As expected, all transgenic plants produced detectable levels of both pheromones, but only in the case of SxPv1.2, Z11-16OAc and Z11-16OH accumulated at similar levels. In all the SxPv1.2 samples, the higher Z11-16OAc accumulation seems to result in lower precursor alcohol levels, compared with equivalent SxPv1.0 samples. Both insect pheromones are produced at higher levels in adult plant leaves (Figure 4(b)) when compared with young plant leaves (Figure 4(a)) and roots (Figure 4(c)). All pheromone-producing plants showed considerably reduced plant size; however, the growth penalty was significantly more pronounced in plants accumulating mainly Z11-16OH, whereas the conversion into the acetate form in SxPv1.2 seems to partially relieve the dwarf phenotype. Interestingly, both SxPv1.0 and SxPv1.2 plants showed similar morphology, with short petioles curved upwards and resulting in a compact “cabbage-like” characteristic shape (Figure 4(d)). Both SxP lines showed early senescence symptoms, with premature and progressive yellowing, which led, in the case of SxPv1.0, to the premature death of the plants soon after the fruits set.

### 2.4. The Plant Volatilome Is Affected by Pheromone Production

**Figure 5:**
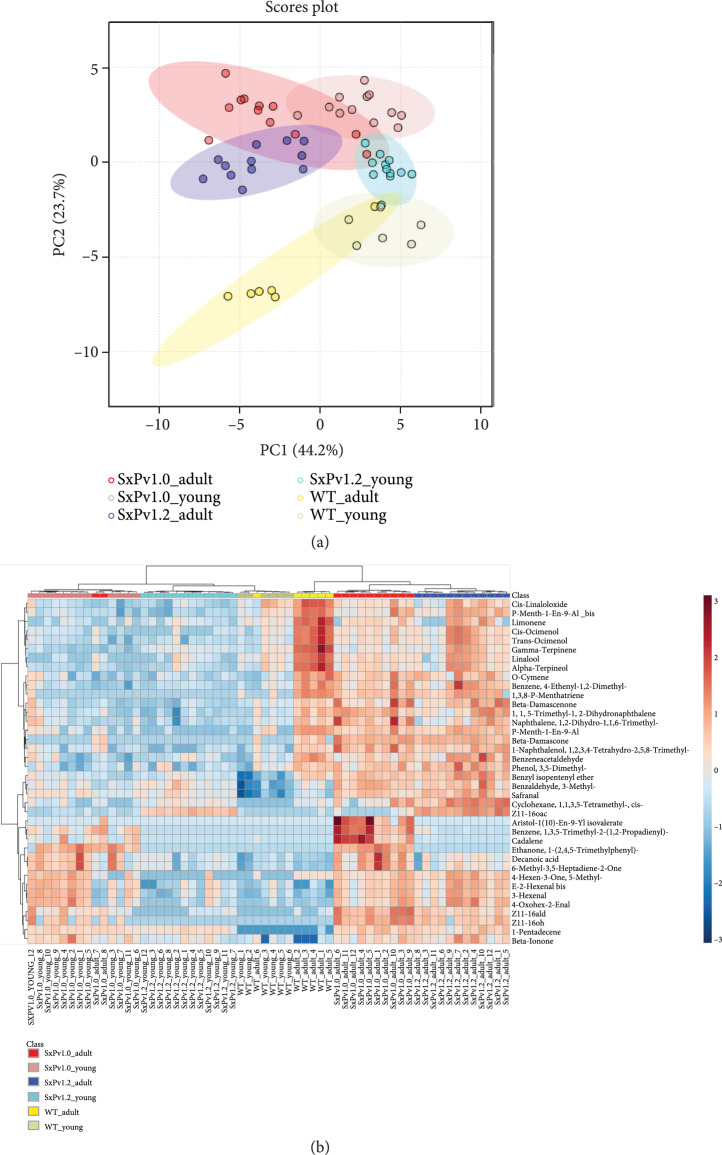
Untargeted analysis of the volatilome of SxPv1.0 and SxPv1.2 and of WT *N. benthamiana*. (a) Principal component analysis and (b) hierarchical clustering and heatmap representation (obtained using Ward’s minimum variance method and Euclidean distance) of the composition of the volatilome of SxPv1.0, SxPv1.2, and wild-type *N. benthamiana* leaves. Twelve individuals for each SxP genotype and six WT plants were analyzed at two developmental stages, young (4 weeks old) and adult (7 weeks old).

A nontargeted analysis of the plant volatile profiles was undertaken to understand the influence of the engineered pheromone pathway on the volatilome. The analysis included leaf samples of 12 young and 12 adult plants from the progeny of SxPv1.0 5_1_7, SxPv1.2_4, and the wild type. The principal component analysis score plot based on the volatile profile showed clustering of the samples based on the different sample classes (Figure 5(a)). The first component accounted mainly for differences in leaf age, whereas the second principal component separated samples according to their genotype. Remarkably, SxPv1.2 samples have, according to both components, intermediate characteristics between the WT and SxPv1.0. The greater separation of SxPv1.0 and the WT probably reflects the more deleterious phenotypic effects experienced by lines accumulating higher Z11-16OH levels.

A clustered heatmap provides interesting visual information on the volatile leaf profiles of SxP plants (Figure 5(b)). The clustering reproduces with few exceptions the different classes, indicating that each genotype and each developmental stage produce a differential and characteristic blend of VOCs. In addition to the pheromone compounds themselves, which are clearly clustered in their respective groups, all SxP plants differentially accumulate other fatty-acid-derived volatile compounds (e.g., (*E*)-2-hexenal and 1-pentadecene), indicating a general activation of this metabolic pathway. Some compounds are characteristic of the adult stage, independently of the genotype. This is the case for some apocarotenoids, such as *β*-damascenone and *β*-damascone, and some phenylalanine-derived compounds, such as o-cymene and phenylacetaldehyde. Other VOCs, such as monoterpenoids (*α*-terpineol, linalool, limonene, and ocimenol), are markedly more abundant in WT than in SxP leaf tissues, with a gradient in which SxPv1.2 shows intermediate features between the wild type and the SxPv1.0 genotype. On the other hand, SxPv1.0 plants display a specific subset of volatile compounds (including the sesquiterpene cadalene) that accumulate at increasing levels at the adult stage. Z11-16Ald is detectable in both SxPv1.0 and SxPv1.2, although its levels are higher especially in leaves from adult SxPv1.0 plants, correlating with higher Z11-16OH production. In SxPv1.2 plants, in which Z11-16OH is partially converted to Z11-16OAc, Z11-16Ald is present at lower levels. Z11-16OAc is, instead, clearly restricted to SxPv1.2. The levels of all three pheromones increase with plant age.

### 2.5. Pheromone Identification and Determination of Its Biological Activity

**Figure 6:**
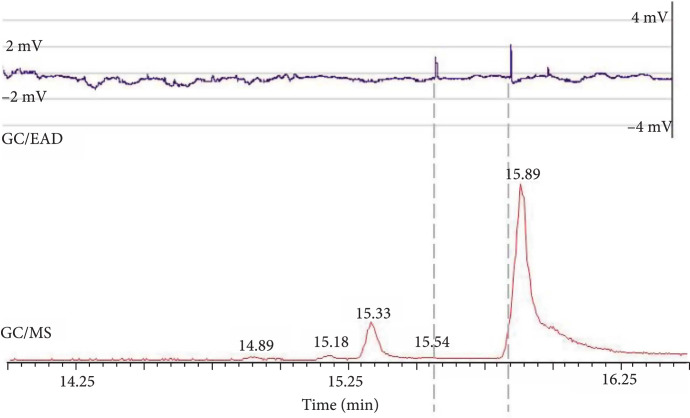
Electrophysiological activity. GC/MS chromatogram and EAD recording showing *Sesamia nonagrioides* male antenna response to the biosynthetic Z11-16OH (retention time=15.89 min). Other compounds contained in the tested fraction were able to interact with the antennal receptors and triggered antennal responses (e.g., retention time 15.54). The GC-MS/EAD run was performed in a GC column ZB-5MS fused silica capillary column (30 m×0.25 mm i.d.×0.25 μm; Phenomenex Inc., Torrance, CA).

Samples of Z11-16OH, Z11-16OAc, and Z11-16Ald were synthesized and characterized by GC/MS and nuclear magnetic resonance (NMR) to have analytical standards of the biosynthetic targets. Additionally, to provide unequivocal identification of the plant-made compound, hexane extracts of 120 g of SxPv1.2 leaves were purified by gravity column chromatography after solvent evaporation, and a 2 mg sample of the purest fractions of the biosynthesized alcohol (Z11-16OH) was also analyzed using NMR. The purity assigned by GC/MS was ca. 78% (Figure S3), and the data extracted from the main signals of both ^1^H and ^13^C NMR spectra were fully consistent with those obtained for the synthetic sample of Z11-16OH, confirming the structure and the *cis*-configuration of the double bond (Figures S4-S5). Further confirmation of the biological activity was provided by electrophysiological analysis. Hexane extracts of SxPv1.2 leaves were fractionated by column chromatography, and the fractions were analyzed by GC/MS. Those fractions that mainly contained Z11-16OH were gathered and employed in electroantennography (EAG) assays with *Sesamia nonagrioides* male moths. The plant-made pheromone was active, since the EAG probe registered significant antennal depolarizations when Z11-16OH reached the antennal preparations (Figure 6; retention time 15.89 min). An unidentified compound with a retention time of 15.54 min also elicited an intense response of the antennae but did correspond neither to Z11-16Ald nor to Z11-16OAc.

### 2.6. Quantification of Total Pheromone Content and Release

The total pheromone content and the rate of volatile emission were both quantified in SxPv1.2 plants. Solvent extraction was carried out in fresh leaves, as well as in leaves stored at -20°C and -80°C to evaluate the total content and the possible loss of pheromone under different storage conditions (Table 1). The Z11-16OH content in leaves was found to be in the range of 0.1 mg g^-1^, (average 111.4±13.7 *μ*g g^-1^), whereas Z11-16OAc accumulated at lower levels (average 11.8±1.3 *μ*g g^-1^). Both pheromones were preserved in frozen leaves, although a part of Z11-16OH could be lost upon storage, although more data will be needed to ensure the actual effect of plant handling and storage temperature. Interestingly, the unbalanced ratio between the two main compounds was compensated when the rate at which pheromones are released to the environment was estimated. As shown in Table 2, an adult SxPv1.2 plant releases on average 79.3±6.3 ng of Z11-16OH and 88.3±11.5 ng of Z11-16OAc per day, as estimated in volatile collection experiments carried out in dynamic conditions. Not unexpectedly, this indicates a much higher volatility of the acetylated moiety. In terms of pheromone release per biomass unit, both compounds are emitted at levels close to 0.01 *μ*g day^-1^ g^-1^ (FW).

**Table 1:** Quantity (*μ*g) of (*Z*)-11-hexadecenol (OH) and (*Z*)-11-hexadecenyl acetate (OAc) extracted from SxPv1.2 individuals by solvent extraction and GC/MS/MS quantification.

Plant	Material	*μ*g OH/g plant	*μ*g OAc/g plant
SxPv1.2 T_1_-3	Fresh leaves	164,9	9,6
SxPv1.2 T_1_-4	Fresh leaves	129,9	8,6
SxPv1.2 T_1_-5	Frozen -20C	78,1	10,5
SxPv1.2 T_1_-6	Frozen -20C	115,1	17,3
SxPv1.2 T_1_-7	Frozen -80C	75,8	11,8
SxPv1.2 T_1_-8	Frozen -80C	104,8	12,9
Mean±se		111.4±13.7	11.8±1.3

**Table 2:** Quantity (ng) of (*Z*)-11-hexadecenol (OH) and (*Z*)-11-hexadecenyl acetate (OAc) released by SxPv1.2 individuals obtained by volatile collection and GC/MS/MS quantification.

Plant	ng collected OH	ng OH/day	ng collected OAc	ng OAc/day
SxPv1.2 T_1_-1	209,3	69,8	193,3	64,4
SxPv1.2 T_1_-2	225,6	75,2	359,4	119,8
SxPv1.2 T_1_-3	222,6	74,2	250,7	83,6
SxPv1.2 T_1_-4	293,9	98,0	256,8	85,6
Mean±se	237.8±19.0	79.3±6.3	265.0±34.6	88.3±11.5

## 3. Discussion

This research was initiated as a Synthetic Biology project in the frame of the iGEM competition, where undergraduate students proposed the use of genetically engineered plants as dispensers of insect sex pheromones. The manufacturing of pheromones and their precursors employing biological factories, such as microbial bioreactors [21, 24] or plant biofactories [18, 19, 25] has become an intensively pursued objective, fuelled by the expected gains in sustainability. Beyond the general biofactory concept, our envisioned long-term approach consists of the design of plants that function as autonomous biodispensers of semiochemicals. A remarkable precedent of this concept was the engineering of wheat plants releasing the alarm pheromone *(E)*-*β*-farnesene as a protective strategy against aphid infestation [11]. Differently to the alarm pheromone concept, which was produced in the crop itself, the proposed biodispenser (originally named as “Sexy Plant”, SxP) is based on the intercropping strategy, where a companion crop, rather than the main crop, is engineered to emit the sex pheromone into the environment. From here, two different strategies can be followed. In a mating disruption strategy [2, 26, 27], the dispensers release pheromones at relatively large quantities, impairing the male’s ability to detect females and therefore disrupting the mating. Oppositely, in mass trapping or attract-and-kill strategies [28], dispensers release pheromones to attract males to traps. This later approach often requires lower pheromone levels to be released into the environment, but in turn requires higher semiochemical specificity (in terms of isomeric purity and exact ratios of the pheromone components), and also some associated equipment to trap and eventually kill the attracted insects.

The genetic engineering of *N. benthamiana* shown here was inspired by the seminal work of Ding et al. [18], where transient expression of various components of moth sex pheromone blends was achieved. Contrary to other insect pests, whose sex pheromones are made of a single, highly specific molecule, as with some mealybugs [29], lepidopteran sex pheromones are often made of more complex blends of fatty-acid derived compounds, many of them shared by several species. Species-specificity in these cases is provided by the precise ratio in the blend. For instance, Krokos et al. [30] tested the response of *Sesamia nonagrioides* (Lefèbvre) males to different blends of pheromone compounds, identifying a 90 : 10 : 5 blend of Z11-16OAc:Z11-16OH:Z11-16Ald as the most effective. This feature makes the genetic design of plant emitters for attract-and-kill strategies in moths extremely challenging, because ensuring the right proportions of the three compounds requires a tight control of several factors, from gene expression to enzymatic activity and differential release ratios. Conversely, mating disruption seems a more attainable objective in terms of heterologous pheromone production since, in many cases, the release of nonattractive incomplete mixtures can disrupt mating as effectively as the complete blend [31]. In this case, however, the main requirement imposed on a biological dispenser is to produce and release sufficient quantities of one or more compounds in the blend. Therefore, the main objective of this work was to understand the biological constraints accompanying the production and release of two of the most representative compounds of lepidopteran pheromone blends in *N. benthamiana* plants. We successfully generated a first generation of transgenic plants (SxPv1.0) producing mainly Z11-16OH. Homozygous SxPv1.0 lines maintained pheromone production up to the T_3_ generation. It should also be noted that basal levels of Z11-16Ald and Z11-16OAc were detected in SxPv1.0, probably produced by endogenous enzymes, since no oxidase was included in this first version of the pathway, and the third enzyme of the route, *EaDAct*, was truncated. The disruption of the *EaDAct* gene in different transgenic lines may be explained by a tendency to recombine with plasmid DNA in the bacterial host. This seems to be the case based on the observation that a small fragment of plasmid origin was found interrupting the coding sequence in the truncated construct. In addition, the distal position of *EaDAct* with respect to the selection marker in SxPv1.0 could have made it more likely that rearrangements in this gene went unnoticed, as they did not affect regeneration on selective media. Although a relatively lower number of regenerants was obtained for SxPv1.1 and SxPv1.2 (8 and 5, respectively), compared to SxPv1.0 (11), this difference is most likely due to contingent factors such as chance, contamination, and even limited access to experimental facilities during the COVID-19 pandemic. The recovery of a single plant producing a blend of Z11-16OH and Z11-16OAc may have been aided by closely linking the previously truncated gene with selection markers to increase the probability of associating positive selection with an intact *EaDAct* gene. With the only exception of the above mentioned SxPv1.2 plant, all SxPv1.1 and SxPv1.2 recovered plants effectively integrated the intact construct but failed to produce measurable levels of pheromone compounds, probably due to silencing or positional effects. This seems to indicate that only certain levels/ratios of the two compounds are compatible with viable plant regeneration and biomass accumulation. Pheromone production in this new single line (now in the T_2_ generation) is also very stable and maintains remarkably homogeneous levels of production. The establishment of SxPv1.0 and SxPv1.2 stable plants has allowed us to study in detail the production levels of the different pheromone components, their relative abundance, and their volatility, together with an in-depth characterization of the accompanying phenotype.

As results of our analysis, two main bottlenecks were identified: the associated growth penalty and the poor release rates of the pheromones to the environment. As highlighted also by Reynolds et al. [32] and later by Xia et al. [19], one of the most significant downsides to the constitutive overexpression of medium-chain fatty acid biosynthesis pathways in plants is the associated developmental abnormalities. These may result from an imbalance caused by diverting metabolic resources from fatty acid metabolism towards the products of interest, and possibly from the toxicity of the end-products. Such toxic effects can hamper plant viability and result in a negative selection pressure against the genotypes with higher pheromone production levels. Interestingly, whereas Xia et al. [19] found strong deleterious effects associated with the production of *(E)*-11-tetradecenoic acid, the same authors regenerated normal plants that accumulated Z11-16CoA, the direct precursor of the volatile pheromones produced here. The fact that the simple addition of a desaturase activity leads to deleterious effects may indicate that Z11-16OH itself is responsible for the toxic effects observed when accumulated in leaves. Furthermore, this toxicity seems partially alleviated when a fraction of Z11-16OH is converted to Z11-16OAc, leading to higher biomass in the case of SxPv1.2.

**Figure 7:**
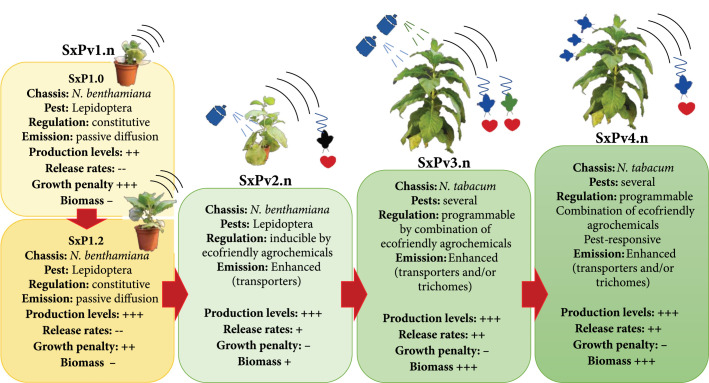
Roadmap for future SxP versions. The bottlenecks identified in SxPv1.n serve as guidance for future iterations in the development of pheromone biofactories. Key improvements in a second generation (SxPv2.n) should include inducible expression of the pheromone pathway to circumvent growth penalties, ideally triggered by environmentally friendly agrochemicals. Additionally, it should increase emission rates, e.g., by coexpressing carrier proteins. Progress towards a third generation (SxPv3.n) would require the transfer of SxPv2.n tools to a related chassis with higher biomass, probably N. tabacum. Other improvements would involve the ability to produce different pheromone “programs” in the same plant, each one triggered by different chemicals, and the selective accumulation of pheromones in glandular trichomes to facilitate their release. Ideally, subsequent iterations (SxPv4.n) should incorporate, among others, the ability to respond directly to the presence of the target pest, as well as additional improvements in the chassis itself that facilitate its use as biosafe emitters in the field (e.g., nonflowering).

Understanding the changes imposed on the leaf volatilome can shed light on the associated phenotypic changes and the possible imbalances produced by the introduction of the recombinant pheromone pathway. We show here that each SxP version has a distinctive volatile profile that differs from wild-type plants primarily by the presence of the pheromones themselves and a few related fatty-acid-derived compounds, which apparently result from endogenous enzyme activities operating on new-to-plant molecules. This seems to be the case for Z11-16Ald (itself a common component of moth pheromone blends) and also for the differential accumulation of other shorter chain fatty acid derivatives such as 1-pentadecene and hexenal. A close look at the clustered analysis shows that wild-type adult *N. benthamiana* tends to produce more monoterpenes (e.g., linalool) and phenolic VOCs (e.g., phenylalanine derivatives) than younger plants. However, this tendency is reduced in SxP plants in general and is even more severe in SxPv1.0 plants. The observed downregulation of the normal volatile components in adult plants could reflect a reduced ability to set up a defence mechanisms. The fact that *N. benthamiana* is considered a generally immune-suppressed species [33, 34] could explain the premature senescence and the early collapse observed in many SxPv1.0 soon after flowering. The reasons behind the changes in the volatile profiles of the different plant lines may depend on a general reduction of plant fitness imposed by the expression of the heterologous pathway or may be due to specific changes affecting development and regulatory mechanisms. Insight into these imbalances may be fruitfully gained by transcriptomic analysis of the different genotypes. A strategy to alleviate deleterious effects would require disconnecting plant growth from pheromone production. This could be done by employing agronomically compatible inducible expression systems for the activation of the pathway, taking advantage of the increasing number of Synthetic Biology tools made available for plants and particularly for *Nicotiana* species [35–37]. Alternatively, the use of a different plant chassis displaying specialized structures, such as glandular trichomes to store potentially toxic pheromone compounds could be advantageous. Glandular trichomes serve as natural biofactories for VOC biosynthesis and release, e.g., in aromatic plant species [38]. A suggested roadmap showing the subsequent SxP version and the improvements they should incorporate in light of the problems encountered in SxPv1 is presented in Figure 7.

The quantification of pheromone tissue accumulation and environmental release in SxPv1.2 leads to interesting considerations. The maximum pheromone accumulation levels measured in SxPv1.2 reached 174.5 *μ*g g^-1^ FW (totalling both alcohol and acetate forms). This is about half of the levels of the precursors reported by Ding et al. [18] in transient experiments (381 *μ*g g^-1^) or by Xia et al. [19] in stable plants (335 *μ*g g^-1^) and may indicate a partial conversion into biologically active forms, or an upper limit for toxicity, especially in the case of Z11-16OH. However, only a small portion of the plant pheromone content can be detected in the environment after a 72 h incubation. Typically, mating disruption strategies require daily release rates between 20 and 500 mg Ha^-1^ day^-1^ [1, 39]. Our data indicates that the maximum release rates per biomass unit are around 20 *μ*g kg plant^-1^ day^-1^; therefore, it would require between 1,000 kg and 25,000 kg of pheromone-producing intercropping biomass per Ha for effective mate disruption. This is obviously not viable for dwarf SxPv1.2 plants, whose average fresh weight is 9.35 g (aerial parts), and it would be still challenging even if plant species with large biomasses are used as bioemitters. Therefore, it is concluded that the improvement in the release rates is an important objective to focus on. In leaves, VOCs are synthesized in mesophyll cells and release takes place through the stomata or cuticle [40]. Emission rates of endogenous VOCs are highly variable and depend on the chemical properties of each molecule. Furthermore, volatility is temperature-dependent, with higher temperatures leading to a more rapid transition from the liquid to the gas phase ([41] and references therein). C16 fatty acid derivatives are indeed semivolatile compounds and, in the absence of specialized structures (like the glandular trichomes described above), active transport may play an important role in their release from mesophyll cells. Active transporters of the adenosine triphosphate-binding cassette (ABC) class are known to be required for the release of at least some volatile components of flower scent in petunia [42]. Pheromone-binding proteins (PBPs) play important roles in binding pheromones and bringing them in contact with receptor complexes in the antennae [43]. Interesting biotechnological approaches have shown that fusing odorant binding proteins with transit peptides allows them to efficiently cross lipid membranes, thus moving odorants to the desired compartments [44]. The use of engineered PBPs or transporters to facilitate pheromone release needs further exploration (Figure 7). The availability of a first version of a live pheromone biodispenser will facilitate the study of transporters and permeability intermediaries and serves as the basis for new design-build-test iterations towards the deployment of efficient SxPs as new components of integrated pest management strategies.

## 4. Materials and Methods

### 4.1. DNA Assembly and Cloning

The basic DNA elements (promoters, coding regions, and terminators) employed for the assembly of multigene constructs (level 0 parts) were designed, synthesised, and cloned using the GoldenBraid (GB) domestication strategy described by Sarrion-Perdigones et al. [45]. Once cloned into a pUPD2 vector, these new DNA elements were verified by enzymatic digestion and by sequencing. Transcriptional units (level 1 parts) were then assembled via multipartite BsaI restriction-ligation reactions from level 0 parts, while level>1 modules were produced via binary BsaI or BsmBI restriction–ligation. All level≥1 parts were confirmed by restriction enzyme analysis. All GB constructs created and/or employed in this study are reported in Table S1, and their sequences are publicly accessible at https://gbcloning.upv.es/search/features. All constructs were cloned using the *Escherichia coli* TOP 10 strain. Transformation was performed using the Mix & Go kit (Zymo Research) following the manufacturer’s instructions. The final expression vectors were transformed into electrocompetent *Agrobacterium tumefaciens* GV3101 C58 or LBA4404 for transient or stable transformations, respectively.

### 4.2. Transient Expression Assays in *Nicotiana benthamiana*

*Agrobacterium tumefaciens* GV3101 cultures harbouring the constructs of interest were grown from glycerol stocks for 2 days to saturation, then refreshed by diluting them 1 : 1000 in LB liquid medium supplemented with the appropriate antibiotics. After being grown overnight, cells were pelleted and resuspended in agroinfiltration buffer (10 mM MES, pH 5.6, 10 mM MgCl_2_ and 200 *μ*M acetosyringone), incubated for 2 hours in the dark, and adjusted to an OD_600_ of 0.1. Equal volumes of each culture were mixed when needed for coinfiltration. A P19 silencing suppressor was included in the mixes to reduce posttranscriptional gene silencing [20]. Agroinfiltration was carried out with a 1 mL needleless syringe through the abaxial surface of the three youngest fully expanded leaves of 4-5-week-old plants grown at 24°C (light)/20°C (darkness) with a 16 : 8 h light : darkness photoperiod. Samples were collected 5 days postinfiltration using a Ø 1.5-2 cm corkborer and snap frozen in liquid nitrogen.

### 4.3. *Nicotiana benthamiana* Stable Transformation

Stable transgenic lines were generated following the transformation protocol of Clemente [46], using *Agrobacterium tumefaciens* LBA4404 cultures with the corresponding plasmids. Briefly, leaves from 4-5-week-old *N. benthamiana* plants grown at 24°C (light)/20°C (darkness) with a 16 : 8 h light : darkness photoperiod were sterilized by washing in a 2.5% sodium hypochlorite solution for 15 minutes, then rinsed in 70% ethanol for 10 seconds and washed 3 times in sterile distilled water for 15 minutes. Leaf discs were then cut using a Ø 0.8-1.2 cm corkborer and transferred to a coculture medium (MS medium supplemented with vitamins, enriched with 1 mg L^-1^ 6-benzylaminopurine and 0.1 mg L^-1^ naphthalene acetic acid). After 24 h on this medium, discs were incubated for 15 minutes in an *Agrobacterium* culture grown overnight to OD_600_ of 0.2 in TY medium (10 g L^-1^ tryptone, 5 g L^-1^ yeast extract, and 10 g L^-1^ NaCl, pH=5.6) supplemented with 2 mM MgSO_4_·7H_2_O, 200 *μ*M acetosyringone and the appropriate antibiotics. After incubation, discs were transferred back to the coculture medium and incubated for 48 h in the dark. Shoots were then induced by transferring to a MS medium supplemented with vitamins, 1 mg L^-1^ 6-benzylaminopurine, 0.1 mg L^-1^ naphthalene acetic acid, and 100 mg L^-1^ kanamycin for selection of transformants. After 2-3 weeks of growth with weekly transfers to fresh media, shoots developing from the calli were isolated and transferred to root-inducing medium (MS supplemented with vitamins and 100 mg L^-1^ kanamycin). All in vitro growth was performed in a growth chamber (16 : 8 h light : darkness photoperiod, 24°C, 60%–70% humidity, 250 *μ*mol m^-2^ s^-1^). Rooted shoots were finally transferred to soil and grown in a greenhouse at 24 : 20°C (light : darkness) with a 16 : 8 h light : darkness photoperiod.

### 4.4. Plant Growth and Sampling

Transgenic SxP seeds were placed in a germination medium (MS with vitamins 4.9 g L^-1^, sucrose 30 g L^-1^, Phytoagar 9 g L^-1^, pH=5.7) supplemented with 100 mg L^-1^ kanamycin for positive transgene selection. Control WT plants were obtained similarly by placing seeds in a non-selective germination medium. WT and kanamycin-resistant seedlings were transferred to the greenhouse a week after germination, where they were grown at 24 : 20°C (light : darkness) with a 16 : 8 h light : darkness photoperiod.

Samples for targeted VOC analysis were collected from the 2^nd^ and 3^rd^ youngest and fully expanded leaves of each plant at the early flowering stage. All samples were collected between 4 and 6 pm, frozen in liquid nitrogen immediately after collection, and ground afterwards. Plant size was also estimated at this stage using a 1-10 scale. WT plants grown in parallel with each batch of transgenic plants were taken as a reference and given a score of 10.

For the comparative study of the SxPv1.0 and SxPv1.2 lines, seeds from SxPv1.0 5_1_7_X (T_2_), SxPv1.2 4_X (T_0_), and WT *N. benthamiana* plants were all sown simultaneously on selective and nonselective MS medium, then transferred to soil and grown in the conditions described above. Leaf samples and pictures were taken at 4 weeks and 7 weeks after transplant, which corresponds to the young and early flowering stages (hereafter, adults), respectively. Roots were collected at the adult stage. All samples were snap-frozen in liquid nitrogen and ground. All samples were analyzed according to the same GC/MS protocol, as described below.

### 4.5. VOC Analysis

50 mg of frozen, ground leaf samples were weighed in a 10 mL headspace screw-cap vial and stabilized by adding 1 mL of 5 M CaCl_2_ and 150 *μ*L of 500 mM EDTA (pH=7.5), after which they were sonicated for 5 minutes. Volatile compounds were captured by means of headspace solid phase microextraction (HS-SPME) with a 65 *μ*m polydimethylsiloxane/divinylbenzene (PDMS/DVB) SPME fiber (Supelco, Bellefonte, PA, USA). Volatile extraction was performed automatically by means of a CombiPAL autosampler (CTC Analytics). Vials were first incubated at 80°C for 3 minutes with 500 rpm agitation. The fiber was then exposed to the headspace of the vial for 20 min under the same conditions of temperature and agitation. Desorption was performed at 250°C for 1 minute (splitless mode) in the injection port of a 6890 N gas chromatograph (Agilent Technologies). After desorption, the fiber was cleaned in a SPME fiber conditioning station (CTC Analytics) at 250°C for 5 min under a helium flow. Chromatography was performed on a DB5ms (60 m, 0.25 mm, 1 *μ*m) capillary column (J&W) with helium as the carrier gas at a constant flow of 1.2 mL min^-1^. For an initial identification of the pheromone peaks, oven programming conditions were 40°C for 2 min, 5°C min^-1^ ramp until reaching 280°C, and a final hold at 280°C for 5 min. Once the target peaks were identified, the oven conditions were changed to an initial temperature of 160°C for 2 min, 7°C min^-1^ ramp until 280°C, and a final hold at 280°C for 6 minutes to reduce the overall running time without losing resolution of the desired compounds. Identification of compounds was performed by the comparison of both retention time and mass spectrum with pure standards (for pheromones) or by comparison between the mass spectrum for each compound with those of the NIST 2017 Mass Spectral library (Supplementary File 1). All pheromone values were divided by the total ion count (TIC) of the corresponding sample for normalization [47].

The quantification of pheromone compounds emitted by plants was carried out by volatile collection in dynamic conditions. Individual plants were placed inside 5 L glass reactors (25 cm high×17.5 cm diameter flask) with a 10 cm open mouth and a ground glass flange to fit the cover with a clamp. The cover had a 29/32 neck on top to fit the head of a gas washing bottle and to connect a glass Pasteur pipette downstream to trap effluents in 400 mg of Porapak-Q (Supelco Inc., Torrance, CA, USA) adsorbent. Samples were collected continuously for 72 h by using an ultrapurified-air stream, provided by an air compressor (Jun-air Intl. A/S, Norresundby, Denmark) coupled with an AZ 2020 air purifier system (Claind Srl, Lenno, Italy) to provide ultrapure air (amount of total hydrocarbons<0.1 ppm). In front of each glass reactor, an ELL-FLOW digital flowmeter (Bronkhorst High-Tech BV, Ruurlo, The Netherlands) was fitted to provide an air push flow of 150 mL min^-1^ during sampling. Trapped volatiles were then extracted with 5 mL pentane (Chromasolv, Sigma-Aldrich, Madrid, Spain), and extracts were concentrated to 200 *μ*L under a nitrogen stream. Twenty microliters of an internal standard solution (TFN, 100 *μ*g/mL in hexane) was added to the resulting extract prior to the chromatographic analysis for pheromone quantification.

### 4.6. Statistical Analysis

For the untargeted volatilome analysis, data preprocessing was performed with Metalign [48]. Peak intensities were calculated for each compound for the SxP and WT samples and for blanks (mock CaCl_2_+EDTA samples), and compounds were included in the analysis if the sample : blank ratio was ≥2 for at least one of the categories (SxPv1.0, SxPv1.2, or WT). The principal component analysis and hierarchical clustering were performed with MetaboAnalyst 5.0 (https://www.metaboanalyst.ca/). After generalized logarithm transformation, data scaling was performed by mean-centering and dividing by the square root of the standard deviation of each variable. Hierarchical clustering was done using Ward clustering algorithm and Euclidean distance measure. Plant size values were analyzed with the nonparametric Kruskal-Wallis test using the Past3 software to determine the significance of plant size differences.

### 4.7. Plant Solvent Extraction

The total quantity of pheromone compounds accumulated in each plant was extracted with toluene (TLN). Plant samples (ca. 3 g), mixed with fine washed sand (1 : 1, plant : sand, w/w), were manually ground with a mortar to aid in tissue breakdown and facilitate the extraction. The resulting material was then transferred to 50 mL centrifuge tubes with 10 mL TLN. The extraction process was assisted by magnetic agitation for 12 h and finally by ultrasound in a Sonorex ultrasonic bath (Bandelin electronic, Berlin, Germany) for 30 min. A 1 mL sample of the resulting extract was filtered through a PTFE syringe filter (0.25 *μ*m). Two-hundred microliters of an internal standard solution (TFN, 100 *μ*g/mL in hexane) was added to the sample prior to the chromatographic analysis for pheromone quantification.

### 4.8. Synthetic Pheromone Samples and Internal Standard Synthesis

A synthetic sample of 1 g of Z11-16OH was obtained following the method described by Zarbin et al. [49]. The sample was carefully purified by column chromatography using silica gel and a mixture of hexane : Et_2_O (9 : 1 to 8 : 2) as an eluent. Evaporation of the solvent of the corresponding fraction generated a sample of 96% purity by GC-FID.

A standard acetylation of Z11-16OH was carried out using acetic anhydride (1.2 eq) and trimethylamine (1.3 eq) as a base in dichloromethane (DCM), generating the corresponding acetate in 95% yield, whose spectroscopical data was fully coincident with that described in the literature [49]. Oxidation with pyridinium chlorocromate of a 100 mg sample of Z11-16OH was carried out following the method described by Zakrzewski et al. [50] generating 62 mg (60%) of Z11-16Ald, whose spectroscopical data was fully coincident with that described in the literature [50].

Due to the abundance of compounds structurally related to the pheromone in the biological samples, a straight chain fluorinated hydrocarbon ester (heptyl 4,4,5,5,6,6,7,7,8,8,9,9,9-tridecafluorononanoate; TFN) was selected as the internal standard to improve both sensitivity and selectivity for MS/MS method optimization. TFN was synthesized as follows: to a solution of 4,4,5,5,6,6,7,7,8,8,9,9,9-tridecafluorononanoic acid (500 mg, 1.3 mmol) in DCM, oxalyl chloride was added. After 60 min of continuous stirring, the solvent was removed under vacuum. The residue was redissolved in dry DCM (15 mL) and 1-heptanol (0.26 mL, 1.5 mmol), followed by addition of triethyl amine (0.31 mL, 3 mmol) at room temperature, and the resultant solution was refluxed for 24 h. After this period, 15 mL of DCM was added and the solution was successively washed with HCl (1 M, 20 mL), NaHCO_3_ (sat., 20 mL), and brine (15 mL) and dried with anhydrous MgSO_4_. The solution was filtered, and the residue was purified by column chromatography (silica gel; eluent: 1% Et_2_O/hexane) to yield heptyl 4,4,5,5,6,6,7,7,8,8,9,9,9-tridecafluorononanoate (281 mg, 45%), as a colorless oil of 95% of purity estimated by GC-FID. MS (70 eV, m/z): 393 (10%), 375 (40%), 373 (5%), 132 (10%), 98 (30%), 83 (15%), 70 (100%), 69 (70%), 57 (90%), and 56 (90%).

### 4.9. Plant Extract Preparation for Biosynthetic Pheromone Characterization

120 g of a pool of 10 T_1_ SxPv1.2 plants (whole aerial portion of the plant) were mixed with fine washed sand (1 : 1, plant : sand, w/w) and were manually ground with a mortar to aid tissue breakdown and facilitate the extraction. The resulting material was then transferred to a 1 L Erlenmeyer flask, and 400 mL of hexane were added. The extraction process was assisted by magnetic agitation for 12 h. After this time, the mixture was filtered, and the filtrated was concentrated in a rotary evaporator. The residue (ca. 2 g) was chromatographed in a gravity column (30 cm×1.5 cm) using silica gel (50 g) as the stationary phase and a mixture of hexane : Et_2_O (9 : 1) as the solvent. 60 fractions of ca. 3 mL were collected and analyzed by thin layer chromatography and GC/MS. Those fractions containing biosynthetic Z11-16OH were selected and those containing mainly biosynthetic Z11-16OH where mixed, and the solvent was rotary evaporated, generating 2 mg of material. The ^1^H and ^13^C NMR spectrum of the isolated biosynthetic Z11-16OH was recorded by a Bruker 600 Ultrashield Plus spectrometer (Bruker, Billerica, MA) at a frequency of 600 MHz, using CDCl_3_ as the solvent and tetramethylsilane (TMS) as the internal standard.

### 4.10. Pheromone Quantification

The quantification of the pheromone compounds was carried out by gas chromatography coupled to mass spectrometry (GC/MS/MS) using a TSQ 8000 Evo triple quadrupole MS/MS instrument operating in SRM (selected reaction monitoring) mode using electron ionization (EI +), coupled with a Thermo Scientific TRACE 1300 gas chromatograph (GC). The GC was equipped with a ZB-5MS fused silica capillary column (30 m×0.25 mm i.d.×0.25 μm; Phenomenex Inc., Torrance, CA). The oven was held at 60°C for 1 min then was raised by 10°C min^-1^ up to 110°C, maintained for 5 min, raised by 10°C min^-1^ up to 150°C, maintained for 3 min and finally raised by 10°C min^-1^ up to 300°C held for 1 min. The carrier gas was helium at 1 mL min^-1^. For each compound, pheromone components (Z11-16OH and Z11-16OAc), and the internal standard (TFN), the MS/MS method was optimized by selecting the precursor ion and the product ions that provided the most selective and sensitive determinations (Table S2).

The amount of pheromone and the corresponding chromatographic areas were connected by fitting a linear regression model, y=a+bx, where y is the ratio between pheromone and TFN areas and x is the amount of pheromone.

### 4.11. Plant Extract Fractionation for Electroantennography Assays

10 g of T_1_ SxPv1.2 plants (whole aerial portion of the plant) were mixed with fine washed sand (1 : 1, plant : sand, w/w) and were manually ground with a mortar to aid tissue breakdown and facilitate the extraction. The resulting material was then transferred to 50 mL centrifuge tubes with 40 mL TLN. The extraction process was assisted by magnetic agitation for 12 h and finally by ultrasounds in a Sonorex ultrasonic bath (Bandelin electronic, Berlin, Germany) for 60 min. After this time, the mixture was filtered off and the filtrated was concentrated in a rotary evaporator. The residue (ca. 0.2 g) was chromatographed in a gravity column (17 cm×1 cm) using silica gel (15 g) as a stationary phase and a mixture hexane : Et_2_O (9 : 1) as a solvent. Twenty-five fractions of ca. 2 mL were collected and analyzed by thin layer chromatography and GC-MS. Fractions 17-20 containing biosynthetic Z11-16OH were selected and combined for electroantennography assays.

### 4.12. Electroantennography Assays for Evaluating Moth Response to Biosynthetic Pheromone

Starter specimens of *Sesamia nonagrioides* (Lefèbvre) (Lepidoptera: Noctuidae) were collected from infested rice (*Oryza sativa*) plants in paddy fields located in Valencia (Spain). These were maintained on the stems until pupae were obtained and the progeny of the resulting adults was reared on an artificial diet [51]. Pupae were sexed using a stereomicroscope and males were kept separated from females in different chambers under an L16:D8 regime at 25±2°C and 60% relative humidity.

The electrophysiological response of *S. nonagrioides* males to the biosynthetic Z11-16OH was tested by gas chromatography coupled to mass spectrometry and electroantennography detectors (GC/MS-EAD). For this purpose, 2-3-day-old males were individually placed into test tubes in an ice bath to excise their antenna. Between two and five terminal segments of the antenna were also removed with a scalpel. The antenna was mounted between silver wire electrodes impregnated with conductive electrode gel (Spectra 360, Parker Laboratories, Inc., Fairfield, NJ, USA), to increase the electrical contact. A humidified and carbon-filtered airflow (50 mL/min) was directed continuously over the antenna preparation through a glass L-tube placed at less than 2 cm distance. The flow was delivered by a Syntech CS-55 stimulus controller (Ockenfels Syntech GmbH, Kirchzarten, Germany). A pore-sized opening in the elbow part of the L-tube allowed the introduction of the distal part of a fused-silica restrictor connected to the GC apparatus (Clarus 600 GC/MS, Perkin Elmer Inc., Wellesley, PA). The effluent of the GC column (ZB-5MS fused silica capillary column (30 m×0.25 mm i.d.×0.25 μm; Phenomenex Inc., Torrance, CA) was split 1 : 40 for simultaneous detection between the MS and the EAD apparatus. A Swafer S splitter (Perkin Elmer Inc., Wellesley, PA) was employed for this purpose. The GC-MS/EAD run was performed with the SxPv1.2 extract fraction containing the biosynthetic Z11-16OH obtained as described above. The GC oven temperature was programmed at 120°C for 2 min, then raised to 200°C at 10°C/min and finally from 200°C to 280°C (held for 10 min) at 5°C/min. The EAG responses were recorded with a Syntech IDAC 2 acquisition controller, and GC-EAD 32 (v. 4.3) software was employed for data recording and acquisition (Ockenfels Syntech GmbH, Kirchzarten, Germany).

## Data Availability

All data is freely available upon request and will be deposited to the FAIRDOMHub database in the frame of the Era-CoBiotech SUSPHIRE project.

## Additional Points

Current address of Alfredo Quijano-Rubio is: Institute for Protein Design, University of Washington, Seattle, WA, USA. Current address of José Gavalda-García is: Interuniversity Institute of Bioinformatics, Brussels, Belgium. Current address of Lucía Estellés is: CNAG-CRG, Centre for Genomic Regulation (CRG), The Barcelona Institute of Science and Technology, Baldiri Reixac 4, Barcelona 08028, Spain. Current address of Alba Rubert is: Vrije Universiteit, Amsterdam, The Netherlands.

## Supplementary Materials

Supplementary 1 Supplementary Data 1. Table S1: GoldenBraid Phytobricks created and used in this study. Table S2: optimized values of the MS/MS parameters for each target compound. Table S3: primers created and used in this study for testing the integrity of the EaDAct gene in SxPv1.0 plants. Figure S1: transient expression in Nicotiana benthamiana of the moth pheromone synthetic pathway. Figure S2: gel electrophoresis of the PCR results with gDNA and cDNA from SxPv1.0 T2 plants. Figure S3: GC/MS of biosynthetic Z11-16OH. Figure S4: ^1^H NMR of biosynthetic Z11-16OH. Figure S5: ^13^C NMR of biosynthetic Z11-16OH.

Supplementary 2 Supplementary File 1: data of principal component analysis and heatmap representation.
