# Supplementary material for: Production of Volatile Moth Sex Pheromones in Transgenic Nicotiana benthamiana Plants
Source: Biodes Res. 2021 Oct 12;2021:9891082. doi: 10.34133/2021/9891082 (PMC10521740; doi:10.34133/2021/9891082)
Supplement: Supplementary 1 — Supplementary Data 1. Table S1: GoldenBraid Phytobricks created and used in this study. Table S2: optimized values of the MS/MS parameters for each target compound. Table S3: primers created and used in this study for testing the integrity of the EaDAct gene in SxPv1.0 plants. Figure S1: transient expression in Nicotiana benthamiana of the moth pheromone synthetic pathway. Figure S2: gel electrophoresis of the PCR results with gDNA and cDNA from SxPv1.0 T2 plants. Figure S3: GC/MS of biosynthetic Z11-16OH. Figure S4: 1H NMR of biosynthetic Z11-16OH. Figure S5: 13C NMR of biosynthetic Z11-16OH. [file 9891082.f1.docx]

**Supplementary data**

**Table S1.** GoldenBraid Phytobricks created and used in this study.

| **GB ID** | **Name** | **Description** |
| --- | --- | --- |
| GB1018 | HarFAR CDS | CDS of *Helicoverpa armigera* farnesyl reductase (accession number JF709978) |
| GB1019 | AtrΔ11 CDS | CDS of *Amyelois* *transitella* Δ11-desaturase (accession number JX964774) |
| GB1020 | EaDAct CDS | CDS of the *Euonymus* *alatus* acetyltransferase (accession number GU594061) |
| GB1021 | P35S:HarFAR:T35S | TU for the constitutive expression of *Helicoverpa armigera* farnesyl reductase |
| GB1022 | P35S:EaDAct:T35S | TU for the constitutive expression of *Euonymus alatus* acetyltransferase |
| GB1023 | P35S:AtrΔ11:T35S | TU for the constitutive expression of *Amyelois transitella* Δ11-desaturase |
| GB1024 | P35S:AtrΔ11:T35S-P35S:HarFAR:T35S | Module for the constitutive expression of *Amyelois transitella* Δ11-desaturase and *Helicoverpa armigera* farnesyl reductase |
| GB1025 | P35S:AtrΔ11:T35S-P35S:HarFAR:T35S-SF-P35S:EaDAct:T35S | Module for the constitutive expression of *Amyelois transitella* Δ11-desaturase, *Helicoverpa armigera* farnesyl reductase and *Euonymus alatus* acetyltransferase |
| GB1491 | Tnos:NptII:Pnos-P35S:DsRed:Tnos-P35S:AtrΔ11:T35S-P35S:HarFAR:T35S-P35S:EaDAct:T35S | Module for the constitutive expression of *Amyelois transitella* Δ11-desaturase, *Helicoverpa armigera* farnesyl reductase and *Euonymus alatus* acetyltransferase, together with NptII and DsRed marker genes |
| GB3534 | Omega1_AtrD11+HarFAR+EaDAct+SF | Module for constitutive expression of the Sexy Plant enzymes |
| GB3535 | Omega2_DsRed+nptII | Module for constitutive expression of DsRed and nptII selection genes |
| GB3536 | Alpha1_AtrD11+HarFAR+EaDAct+SF+DsRed+nptII | Module for stable transformation of Sexy Plant genes |
| GB3537 | omega1_DsRed+SF | Construct made to have the DsRed selection gene in an omega plasmid |
| GB3538 | omega2_AtrD11+HarFAR+EaDAct+nptII | Module for constitutive expression of the Sexy Plant genes and the nptII selection marker |
| GB3539 | Alpha1_DsRed+SF+AtrD11+HarFAR+EaDAct+nptII | Module for stable transformation of Sexy Plant genes, flanked by selection markers at both sides |

**Table S2.** Optimized values of the MS/MS parameters for each target compound.

| Compound | Transition^1^ | Precursor ion (*m/z*) | Product ion (*m/z*) | Collision energy (eV) |
| --- | --- | --- | --- | --- |
| TFN | 1* | 393 | 375 | 5 |
|  | 2 | 375 | 263 | 10 |
| Z-11-C16:OH  Z-11-C16:OAc | 1 | 82 | 67 | 5 |
|  | 2 | 95 | 67 | 10 |
|  | 3* | 96 | 54 | 10 |
|  | 4 | 96 | 81 | 5 |

^1^ Transitions denoted with (*) were the ones employed to obtain the corresponding chromatographic areas. The others were monitored for confirmatory purposes to have increased selectivity when several peaks appear near to each peak retention time.

**Table S3.** Primers created and used in this study for testing the integrity of the EaDAct gene in SxPv1.0 plants.

| **Primer pair name** | **Primer sequences (Fw; Rv)** | **Amplicon fragment size** |
| --- | --- | --- |
| Full | TGCTTCGGCTTCTTTCACTT; GCGATAATGGCAGGGAAGTA | 637 bp |
| CDS | TTGTCTCCCCATAACAATTA; CATGACAACAATATATCACG | 200 bp |
| CHI | CCGGATAGGAATTGGCTAAGATCAT; TGCATTTCACATGCTTGAGTTGACC | 1400 bp |

**(a) (b)**


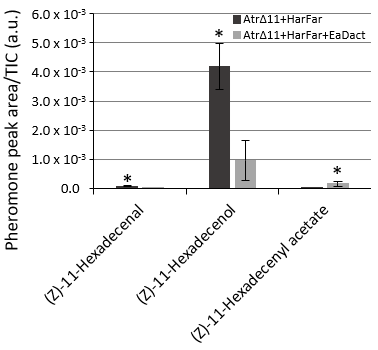

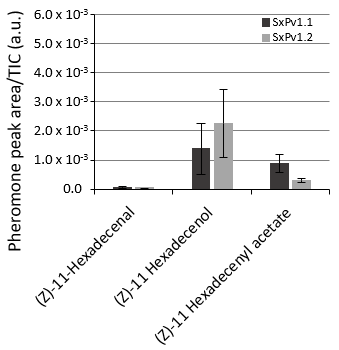


**(c)**


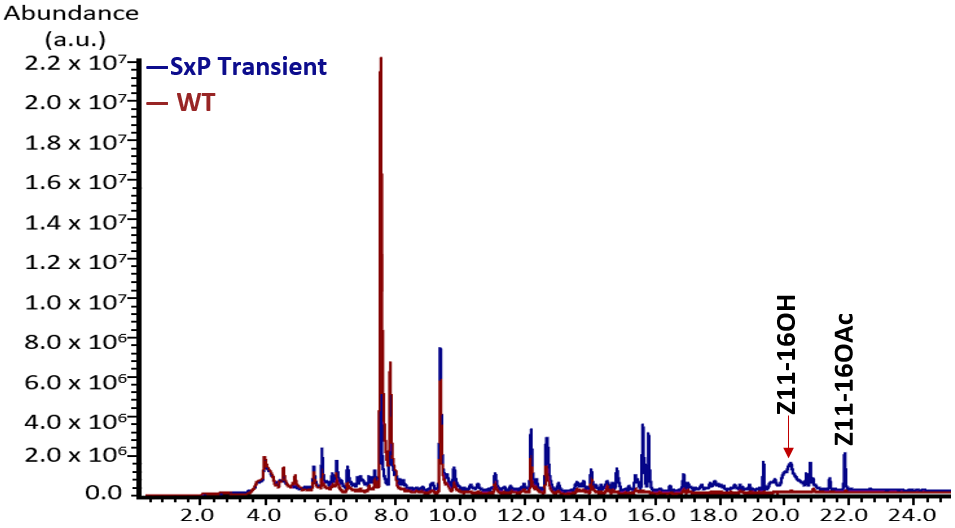


**Figure S1.** Transient expression in *Nicotiana benthamiana* of the moth pheromone synthetic pathway. (a) GC/MS quantification of the three pheromones when the three enzymes were transiently expressed, compared to the expression of only the first two enzymes (*AtrΔ11*, *HarFAR*). A 40x increase in acetate levels was observed when *EaDAct* was present, followed by a 4x decrease in the alcohol levels and a 3x decrease of aldehyde levels (T-test, α=0.05). (b) Pheromone levels obtained transiently with the SxPv1.1 and SxPv1.2 constructs. Error bars represent the ± SE of the measurements of three different leaf samples. (c) A representative chromatogram of a *Nicotiana benthamiana* plant agroinfiltrated with the SxPv1.2 construct (blue line) and a WT plant (red line).

**(a)**


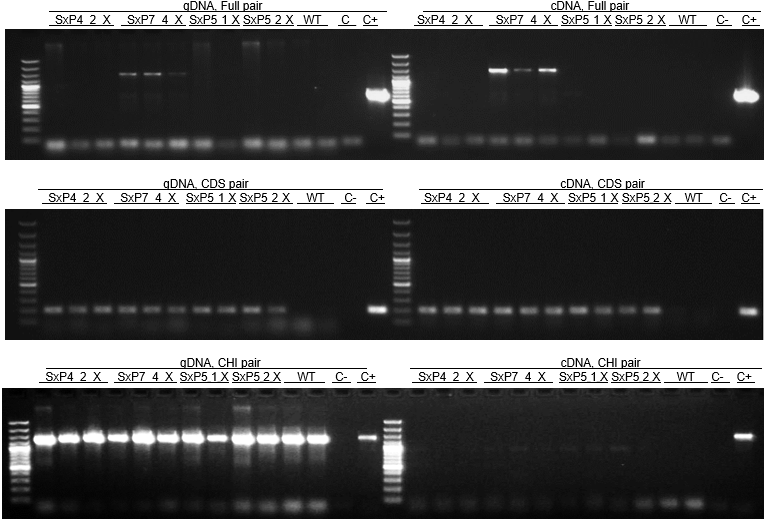


**(b)**

GGACGGTATGGCATTATGATCGCAACTTTGATATCAGCTCATTCATCTTATCAAGTATAACGGGATTTTTTCTAGCTTGGCTTACCACCTTTAAGGTCATTAGCTTTGCATTCGATCAAGGCCCATTATACCCATTACCTCAGAATCTTCTTCATTTTATCTCAATTGCTTGTCTCCCCATAACAATTAAAAGAAATCCAAGCCCAAAATTGAAATCTACAACTAATCCATCACCAATCAGTCATCTTCTTAAAAAAGCCTTTATGAGTTTTCCATCCAAGGTGCTATTCCATTGGGTTATCGCTCATCTGTACCAATACAAAAAATATATGGACCCGAACGTTGTGCTCGTGATATATTGTTGTCATGTTTACGTGGGTGATGCTGCCAACTTACTGATTTAGTGTATGATGGTGTTTTTGAGGTGCTCCAGTGGCTTCTGTTTCTATCAGCTGTCCCTCCTGTTCAGCTACTGACGGGGTGGTGCGTAACGGCAAAAGCACCGCCGGACATCAGCGCTATCTCTGCTCTCACTGCCGTAAAACATGGCAACTGCAGTTCACTTACACCGCTTCTCAACCCGGTACGCACCAGAAAATCATTGATATGGCCATGAATGGCGTTGGATGCCGGGCAACAGCCCGCATTATGGGCGTTGGCCTCAACACGATTTTACGTCACTTAAAAAACTCAGGCCGCAGTCGGTAACCTCGCGCATACAGCCGGGCAGTGACGTCATCGTCTGCGCGGAAATGGACGAACAGTGGGGCTATGTCGGGGCTAAATCGCGCCAGCGCTGGCTGTTTTACGCGTATGACAGTCTCCGGAAGACGGTTGTTGCGCACGTATTCGGTGAACGCACTATGGCGACGCTGGGGCGTCTTATGAGCCTGCTGTCACCCTTTGACGTGGTGATATGGATGACGGATGGCTGGCCGCTGTATGAATCCCGCCTGAAGGGAAAGCTGCACGTAATCAGCAAGCGATATACGCAGCGAATTGAGCGGCATAACCTGAATCTGAGGCAGCACCTGGCACGGCTGGGACGGAAGTCGCTGTCGTTCTCAAATCGTGGGAGCTGCATGACAAAGTCATCGGGCATTATCTGAACATAAAAACACTATCATAGTGGAGTCATTACCCGTTTACGTGATGTGAATATCAGTTGGAGTCTTTGCGCCACTTTAGCAGAGTTCCTTTGTGGTTTTGATGTTGATCCTCAGTTCAAGGAACCATATTTAGCCACTTCTTTGCAGGACTTCTGGGGACGGAGGTGGAACATTATAGTCTCTTCAGTCTTGAGGTCTACTGTTTATGCACCGACGCGTAACATCGCTCAACTCTTCGTCCGTTTTT

**Figure S2**. Gel electrophoresis of the PCR results with gDNA and cDNA from SxPv1.0 T_2_ plants of the lines SxP4_2_X, SxP7_4_X and two representative plants for SxP5_1_X and SxP5_2_X (a) and sequence obtained from the 1.5kb band observed in SxP7_4 samples for the Full primer pair (b). Highlighted is the unknown sequence found in the middle of the EaDAct coding sequence. Blast results suggest this sequence could be due to T-DNA re-organizations (data not shown).

**
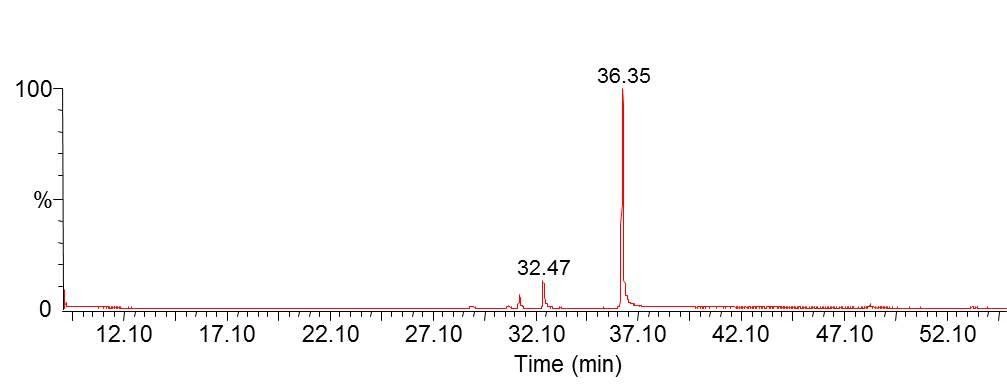
**

**Figure S3**. GC/MS of biosynthetic Z11-16OH.


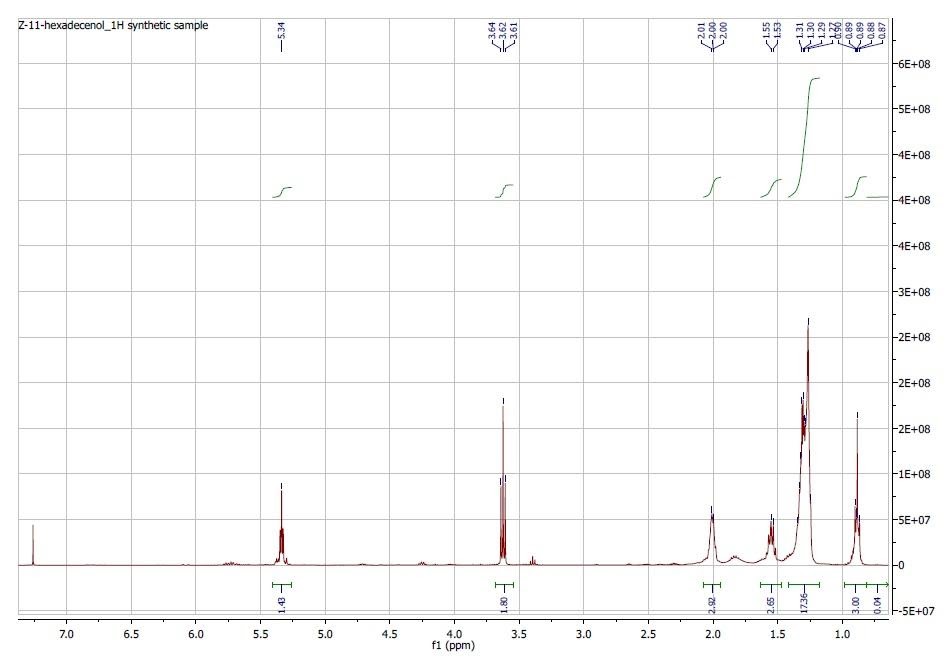


**Figure S4**. ^1^H NMR of biosynthetic Z11-16OH.  Despite our observation that spectroscopical data were fully consistent with those reported by Zarbin et al. [49], some ^13^C signal described were wrongly assigned or duplicated in the original paper, so we list here a corrected version. Spectroscopical data for Z11-16OH: ^1^H NMR (400 MHz, CDCl3) δ: 0.89 (t, J=6.8 Hz, 3H); 1.22–1.35 (m, 18H); 1.48–1.60 (m, 2H); 1.95–2.05 (m, 4H); 3.62 (t, J=6.8, 2H); 5.30–5.38 (m, 2H).

**
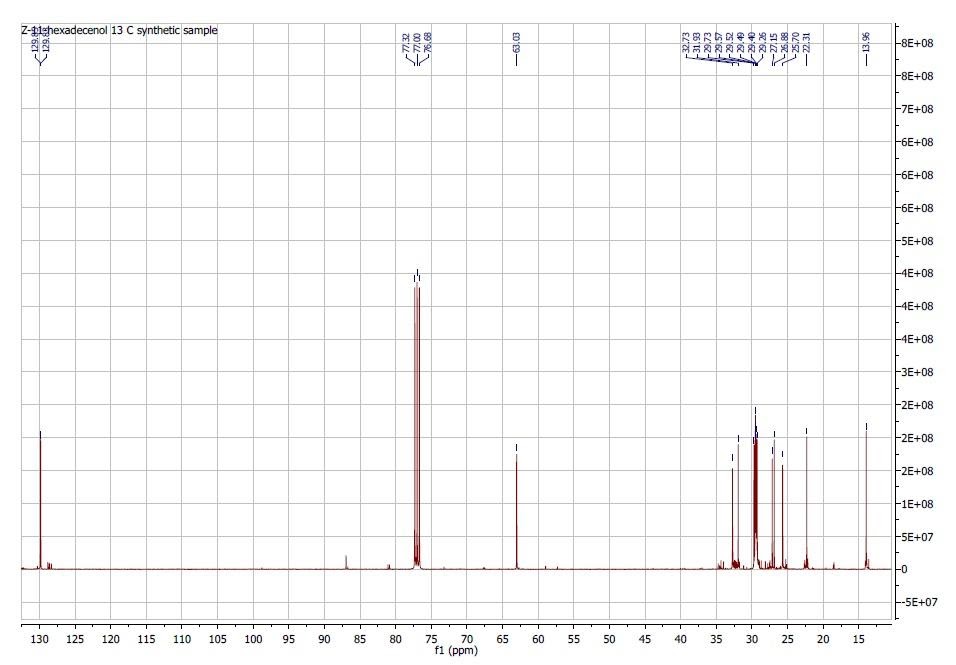
**

**Figure S5**. ^13^C NMR of biosynthetic Z11-16OH.  Despite our observation that spectroscopical data were fully consistent with those reported by Zarbin et al. [49], some ^13^C signal described were wrongly assigned or duplicated in the original paper, so we list here a corrected version. ^13^C NMR (100 MHz, CDCl3) δ: 13.96, 22.31, 25.70, 26.88, 27.15, 29.26, 29.40, 29.49, 29.52, 29.57, 29.73, 31.93, 32.73, 63.03, 129.85 (2C). MS (70 eV, m/z): 222 (M^+^, 3%), 152 (1%), 137 (4%), 123 (9%), 109 (18%), 96 (48%), 82 (63%), 67 (54%), 55 (100%).
